# Subacute (De Quervain's) thyroiditis presenting as painful thyroid nodule suspicious of papillary thyroid carcinoma: Case report

**DOI:** 10.1016/j.ijscr.2022.107329

**Published:** 2022-06-23

**Authors:** Mohamed S. Al Hassan, Walid El Ansari, Mahir Petkar, Sabir A. Al Sharani, Abdelrahman Abdelaal

**Affiliations:** a Department of General Surgery, Hamad General Hospital, Doha, Qatar; b Department of Surgery, Hamad General Hospital, Doha, Qatar; c College of Medicine, Qatar University, Doha, Qatar; d Weill Cornell Medicine–Qatar, Doha, Qatar; e Department of Laboratory Medicine and Pathology, Hamad General Hospital, Hamad Medical Corporation, Doha, Qatar; f Department of Clinical Imaging, Hamad General Hospital, Doha, Qatar

**Keywords:** Subacute thyroiditis, Thyroid antibodies, COVID-19, Nodule, Granulomatous thyroiditis, Neck pain

## Abstract

**Introduction:**

We report a case of subacute thyroiditis (SAT) in a 29 -year-old female who presented with painful thyroid swelling.

**Presentation of case:**

Patient presented with neck pain, tender neck swelling and generalized fatigue. She had no history of neck or chest radiation or compressive symptoms. Ultrasound (US) imaging reveled bilateral nodules of the thyroid gland. Lymph nodes were unremarkable. FNAC was suspicious for papillary thyroid carcinoma. The patient was discussed at the thyroid multidisciplinary meeting, and after deliberation the decision was to offer the patient the choice of repeating FNAC of the bilateral nodules in one month or to proceed with total thyroidectomy.

**Discussion:**

Despite being aware of the possibility that the final pathology could be benign, the patient insisted on total thyroidectomy, given that her FNAC results were suspicious of papillary thyroid carcinoma, and in order to avoid recurrence of the condition and the pain. Following the patient's preference, total thyroidectomy was performed, and surgery was not straightforward as intraoperatively, there was a diffusely inflamed gland. Histopathology findings revealed benign pathology. Follow up until 1.5 years showed that the patient was satisfied, and with normal voice.

**Conclusion:**

SAT is a painful but potentially self-limiting. In some cases, FNAC findings might be suspicious for papillary thyroid carcinoma. Treatment is mostly conservative, but if the pain is severe and the patient insists on surgery as in our case despite the possibility of the condition being of benign pathology, then surgery should be undertaken.

## Highlights

• Subacute thyroiditis (SAT), is a self-limited but painful disorder of the thyroid.
• Fine needle aspiration and cytology findings might sometimes be suspicious for papillary thyroid carcinoma.
• Treatment is mostly conservative, but if the pain is severe and patient insists on surgery, then surgery should be undertaken.
• In cases of surgery, histopathology reveales benign pathology.

## 1 Background

De Quervain's thyroiditis, also known as subacute thyroiditis (SAT), is a self-limited but painful disorder of the thyroid [1]. It is the commonest granulomatous disease of the thyroid, characterized by inflammation of the gland [2], [3]. SAT usually occurs after viral upper respiratory tract infections [3], and has been associated with e.g., mumps, measles, rubella, coxsackie, and adenovirus, through direct viral toxicity or an inflammatory response against the virus [4]. Recently, evidence on the relationship between COVID-19 and the thyroid has been emerging [5], and evolving knowledge suggests that SARS-CoV-2 can trigger SAT [6].

SAT is the most common cause of a painful thyroid gland [3], as clinically, neck pain is the hallmark, hence the synonym “painful subacute thyroiditis” [7]. Patients may also have fever, general malaise, myalgia, and arthralgia [8]. The incidence of SAT is 12.1/100,000 per year, and frequently, middle aged females are affected, generating a female/male ratio of 5/1 [8], [9], [10], [11]. The average age of onset is 30–50 years [12]. About half the patients have thyrotoxicosis in the acute phase, serum T4 level is disproportionately elevated relative to T3 level, and TSH concentrations are low to undetectable [12]. The condition is usually self-limiting, and non-steroidal anti-inflammatory drugs and beta-blockers or a short course of glucocorticoid therapy for some patients are recommended [9].

We report a case of a middle-aged Jordanian female healthcare worker at our institution. She complained of throat pain and painful neck swelling, and presented with a tender thyroid mass. Ultrasonography (US) showed bilateral thyroid nodules, and a provisional diagnosis of multinodular goiter, possibly subacute thyroiditis was suspected. However, fine needle aspiration and cytology (FNAC) was suspicious of papillary thyroid carcinoma (Bethesda V). We report this case in line with the updated consensus-based surgical case report (SCARE) guidelines [13].

## 2 Presentation of case

A 29 year old Jordanian female was referred from the primary health care center in August 2020 complaining of throat pain and tender neck swelling. She was referred to the Endocrinology department at our institution (Hamad General Hospital, largest tertiary facility in Doha, Qatar), as she had been complaining of increasing neck pain and generalized fatigue since a week. She had history of subclinical hypothyroidism in 2017, and was on levothyroxine 50 μg oral daily. There was family history of hypothyroidism (mother and sisters), but no family history of thyroid cancer. She had no history neck or chest radiation, compressive symptoms, alcohol consumption or smoking. She also had no history of COVID-19 vaccination (vaccines were not approved yet) or infection.

Upon physical examination, the patient had a tender diffuse thyroid swelling with no lymphadenopathy. She was conscious and alert, there was no exophthalmos, and systems examination was unremarkable. The patient had mild tachycardia and tremors. Her laboratory tests suggested hyperthyroidism, manifested by low TSH (0.01 mIU/L), and high FT3 and FT4 (14.4 pmol/L and 49.0 pmol/L respectively). Thyroid peroxidase antibodies were negative and anti-thyroid peroxidase was low (<9 IU/mL). A provisional diagnosis of multinodular goiter with hyperthyroidism secondary to subacute thyroiditis was suspected. She was started on prednisone due to the severity of her symptoms. The symptoms initially improved but then recurred, probably because she required a higher dose of steroids. She also received paracetamol and NSAIDs to control the pain.

**Fig. 1:**
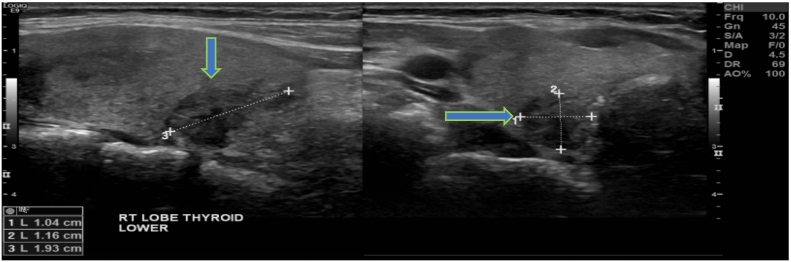
Thyroid ultrasound showing well-defined, mildly lobulated, solid with mixed echogenicity, oval lesion (arrow) lying posterior to the right lobe of the thyroid gland inferiorly.

**Fig. 2:**
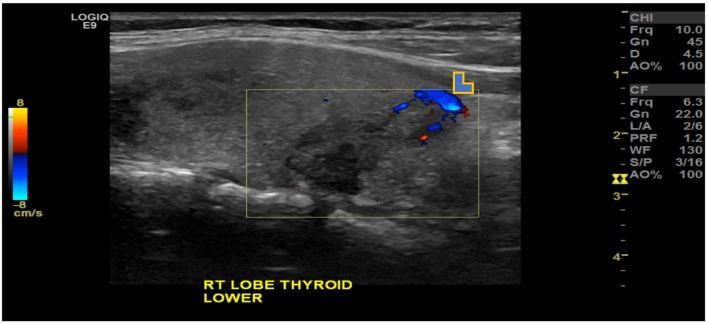
Thyroid ultrasound showing well-defined, mildly lobulated solid lesion posterior to the lower right thyroid lobe displaying peripheral vascularity (arrowhead).

US imaging of the neck revealed a right thyroid nodule (2.1 × 1.4 × 2.2 cm), solid, hypoechoic, with irregular margins, vascular grade 1 and without microcalcifications (Fig. 1, Fig. 2). US also revealed a left thyroid nodule (2.2 × 1.9 × 3.2 cm), solid, hypoechoic, regular margins, vascular grade 1, with microcalcifications. Lymph nodes were unremarkable. Both nodules displayed a high suspicion sonographic pattern as per the American Thyroid Association. Hence, fine needle aspiration and cytology (FNAC) was undertaken for both nodules, and the right and left thyroid showed follicular cells with atypical nuclear grooves in a background of colloid and blood, suspicious of papillary thyroid carcinoma (Bethesda V). The patient was discussed at the thyroid multi-disciplinary team (MDT) meeting, and after deliberation, the decision was to offer the patient the choice of repeating the FNAC of the bilateral nodules in one month or to proceed with total thyroidectomy. The patient opted for total thyroidectomy, given her FNAC results that were suspicious of papillary thyroid carcinoma, and in order to avoid recurrence of the condition and the pain. She was fully aware of the possibility that the final pathology would be benign. Total thyroidectomy was performed. She was discharged after two days and prescribed levothyroxine 125 μg oral daily.

## 3 Surgical technique and intraoperative findings

**Fig. 3:**
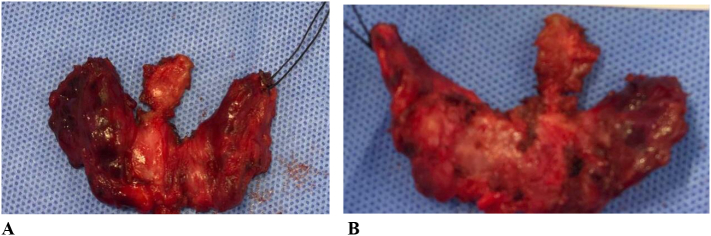
Thyroid specimen showing anterior (A) and posterior (B) views.

Total thyroidectomy was performed in supine position under general anesthesia. A transverse anterior neck incision with the skin crease was undertaken. The right thyroid lobe was identified and dissected, and the middle thyroid vein was ligated and cut. The superior pole vessels were individually ligated and cut, the external branch of superior laryngeal nerve was preserved, and the right recurrent laryngeal nerve identified and preserved. The same was undertaken in the left lobe. Hemostasis was achieved, no drain was left, and the wound was closed in layers. Intraoperatively, there was a diffusely inflamed thyroid that appeared pale and edematous and was adherent to the strap muscles and trachea. The recurrent laryngeal nerves were densely adherent to the gland, however, they were carefully released without any incidents. There were no complications. The specimen (Fig. 3) was sent for histopathology examination.

On day 1 post-surgery, the patient exhibited some stridor and low voice with mild hoarseness. She was assessed by the surgical intensive care unit team and was given dexamethasone 4 mg Q8h and salbutamol nebulization. The patient felt much better, did not require oxygen, spoke in full sentences, her vitals were stable, the wound was clean and dry, and calcium and PTH levels were normal, with no symptoms of hypocalcemia. Her low voice and mild hoarseness lasted about a month after which she returned to her normal voice. Follow up until 1.5 years showed that the patient was satisfied.

**Fig. 4:**
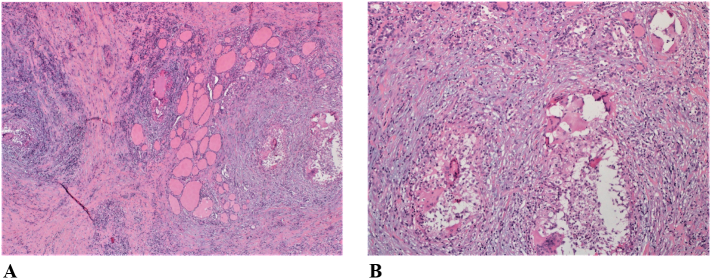
Histopathology showing: (A) Markedly fibrotic thyroid with few residual thyroid follicles, and many non-necrotizing granulomas in the background (H and E ×10); and, (B) higher power view of the granulomas with minimal residual thyroid follicles (H and E ×20).

The histopathology findings revealed a benign pathology. The thyroid capsule was unremarkable. The right lobe was sectioned and revealed an irregular firm nodule (0.9 × 0.2 × 0.1 cm) that occupied the isthmus and extended into the left lobe. Histologically, the gland showed extensive fibrosis, chronic inflammation and minimal residual thyroid follicles with numerous non-necrotizing granulomas in the background. No malignancy was observed. The appearances were those of granulomatous thyroiditis, in keeping with subacute (De Quervain's) thyroiditis (Fig. 4A and B). Grocott stain for fungus and ZN stain for acid-fast bacilli were both negative.

## 4 Discussion

SAT is the most common form of non-autoimmune thyroiditis [14]. It is a self-limited inflammatory thyroid condition caused by viral infection [15]. We report a middle-aged female presenting with a painful thyroid nodule, FNAC findings were suspicious of papillary thyroid carcinoma, but the final histopathology confirmed SAT.

In terms of demography, our patient was a 29 year old female, in agreement with that generally, females are more likely to develop SAT [9], [10], [16], [17]. As for age, typical patients are middle-aged adult females [9], [10], and a review found that, for SAT induced by COVID-19 infection, the youngest patient was 18 years old and the oldest was 85 years old, and typical patients were middle-aged adult females [9].

Regarding the clinical presentation, our patient had complained of increasing neck pain, supporting that neck pain and tenderness are very common with SAT [10], [11], [16], [17], [18]. Our case had generalized fatigue, in agreement with that SAT patients may have general malaise, myalgia, and arthralgia [8], [10], [18]. Likewise, our case had no fever as a main complaint, concurring with that some patients might have fever [8]. The case described in the current report had no history of radiation exposure, supporting other reports [16]. Our patient had mild hyperthyroid symptoms (mild tachycardia and tremors), in agreement with others who observed weight loss, tremor and diarrhoea [16]. However, our case reported family history of hypothyroidism (mother and sisters), in contrast to others who reported no family history of thyroid disease [16].

In terms of thyroid functions, our patient's thyroid functions exhibited hyperthyroidism (low TSH, high FT3 and FT4). These findings support a report of three SAT cases with thyroid functions either within the normal range or compatible with thyrotoxicosis [10]. Our case had normal anti-thyroid peroxidase was <9 IU/mL, in agreement with others [11].

US imaging of the thyroid for the current case showed a right and a left thyroid nodule (both solid and hypoechoic). These findings agree with a report of a SAT patient, where thyroid US revealed three hypoechoic solid nodules in the left and right thyroid lobes [16]. Such hypoechoic areas have also been observed among three SAT cases [10]. Nevertheless, SAT may present as a solitary, painless nodule, hence sometimes the diagnosis may be less clear in such cases [14].

In terms of management, it is symptomatic. This includes non-steroidal anti-inflammatory drugs and beta-blockers during the inflammatory thyrotoxic phase, then levothyroxine if the cases develop symptomatic hypothyroidism, and corticosteroids may be considered for those presenting with moderate-to-severe pain with or without thyrotoxicosis or those who show no response to the initial NSAIDs treatment [11]. Our patient was started on prednisone due to severity of her symptoms, and the pain was controlled with oral paracetamol and NSAIDs. A female SAT case was treated with only a short course of NSAID and her symptoms resolved over 6 weeks, and thyroid function was normal in 8 weeks [11]. For our patient, the MDT decision was to offer the patient the choice of repeating the FNAC of the bilateral nodules in one month, or to proceed with total thyroidectomy. Despite being aware of the possibility that the final pathology could be benign, she insisted on total thyroidectomy, given her FNAC results that were suspicious of papillary thyroid carcinoma, and also to avoid recurrent thyroiditis and further pain. Following the patient's preference, total thyroidectomy was performed, and the surgery on a diffusely inflamed gland due to the recent thyroiditis was not a straight forward one. As systemic long-term glucocorticoid administration has some side effects [19], recently, others undertook an US-guided percutaneous lidocaine-dexamethasone mixture injection outside the thyroid capsule in order to block local nerve conduction [18]. This procedure cured the patient in a week, with no recurrence during the six-month follow up [18].

In connection with etiology, our patient had not received COVID-19 vaccination (vaccines were not approved yet) and also had not contracted a COVID-19 infection at that time. Clinicians need to recognize the possibility of SAT in instances of ongoing or resolved COVID-19 infection [6]. Others reported three female healthcare workers presenting with anterior neck pain and fatigue 4–7 days after SARS-CoV-2 vaccination, with thyroid antibodies and laboratory and imaging findings congruent with SAT [10]. In agreement, post-mortem examinations of individuals who died of COVID-19 showed pathological abnormalities in various organs, including the thyroid gland [20]. Recent studies are exploring the endocrinological effects of COVID-19, and the abnormalities of hypothalamicpituitary-thyroid (HPT) axis, as the thyroid gland is engaged in complicated relationships via hormones and immunomodulation [5], [21], [22]. Two clinical spectra have been observed: that accompanying a severe COVID-19 infection with multi-organ involvement; or an asymptomatic infection, with SAT being the single manifestation or the first presentation [9]. SAT can occur during or after COVID-19. Neck pain, can be mistaken for common sore throat of COVID-19, and persistent tachycardia (despite the clinical amelioration of COVID-19 and absence of other common cardiac causes) should suggest COVID-19-related SAT [5].

## 5 Conclusions

SAT is a painful disorder of the thyroid. The condition is potentially self-limiting and treatment is mostly conservative. In some patients as our case, FNAC findings might be suspicious of papillary thyroid carcinoma. If the pain is severe and the patient insists on surgery as in the current case despite the possibility of the condition being of benign pathology, then surgery should be undertaken, although operating on an inflamed thyroid is not an easy procedure.

## Sources of funding

Nothing to declare.

## Ethical approval

Approved by Medical Research Center, Hamad Medical Corporation reference number (MRC-04-22-368).

## Consent

Written informed consent was obtained from the patient for publication of this case report and accompanying images. A copy of the written consent is available for review by the Editor-in-Chief of this journal on request.

## Author contribution

Mohamed S Al Hassan: Writing - review & editing. Walid El Ansari: study concept, data interpretation, writing the paper, review & editing. Mahir Petkar: Laboratory data, Writing - review & editing. Sabir A. Al Sharani: Ultrasonography data, Writing - review & editing. Abdelrahman Abdelaal: study concept, Writing - review & editing. All authors read and approved the final version.

## Guarantor

Prof. Dr. Walid El Ansari.

## Research registration number

Not first in Man.

## Provenance and peer review

Not commissioned, externally peer-reviewed.

## Declaration of competing interest

Nothing to declare.
